# CdTe and CdSe Quantum Dots Cytotoxicity: A Comparative Study on Microorganisms

**DOI:** 10.3390/s111211664

**Published:** 2011-12-15

**Authors:** Suzete A.O. Gomes, Cecilia Stahl Vieira, Diogo B. Almeida, Jacenir R. Santos-Mallet, Rubem F. S. Menna-Barreto, Carlos L. Cesar, Denise Feder

**Affiliations:** 1 Laboratório de Biologia de Insetos, GBG, Universidade Federal Fluminense—UFF, Niterói, RJ, CEP: 24210-130, Brazil; E-Mail: suzetearaujo@id.uff.br (S.A.O.G.); 2 Laboratório de Transmissores de Leishmanioses, Setor de Entomologia Médica e Forense, IOC-FIOCRUZ, Rio de Janeiro, RJ, CEP: 21040-360, Brazil; E-Mails: ceciliastahl@gmail.com (C.S.V.); jacenir@ioc.fiocruz.br (J.R.S.-M.); 3 Laboratório de Aplicações Biomédicas de Lasers, Departamento de Eletrônica Quântica, Instituto de Física Gleb Wataghin, Universidade Estadual de Campinas, Campinas, SP, CEP: 13083-970, Brazil; E-Mails: dalmeida@ifi.unicamp.br (D.B.A.); lenz@ifi.unicamp.br (C.L.C.); 4 Laboratório de Biologia Celular, IOC-FIOCRUZ, Rio de Janeiro, RJ, CEP: 21040-360, Brazil; E-Mail: rubemb@ioc.fiocruz.br (R.F.S.M.-B.)

**Keywords:** quantum dots, citotoxicity, *Trypanosoma cruzi*, bacteria, microorganisms

## Abstract

Quantum dots (QDs) are colloidal semiconductor nanocrystals of a few nanometers in diameter, being their size and shape controlled during the synthesis. They are synthesized from atoms of group II–VI or III–V of the periodic table, such as cadmium telluride (CdTe) or cadmium selenium (CdSe) forming nanoparticles with fluorescent characteristics superior to current fluorophores. The excellent optical characteristics of quantum dots make them applied widely in the field of life sciences. Cellular uptake of QDs, location and translocation as well as any biological consequence, such as cytotoxicity, stimulated a lot of scientific research in this area. Several studies pointed to the cytotoxic effect against micoorganisms. In this mini-review, we overviewed the synthesis and optical properties of QDs, and its advantages and bioapplications in the studies about microorganisms such as protozoa, bacteria, fungi and virus.

## 1. Introduction

### 

#### Quantum Dots: Optical Properties, Advantages and Bioaplications

Quantum dots (QDs) are semiconductor nanocrystals with optical and electronic properties controlled by their size, morphology and coating. Fluorescent labeling has been the main application of QDs in biology, usually synthesized with II–VI materials such as cadmium telluride (CdTe) or cadmium selenide (CdSe) [1,2]. The strong quantum confinement of 1 to 10 nm nanocrystals changes the QD optical properties of materials [1], allowing the tuning of the fluorescence emission bands [2]. However, the QD fluorescence efficiency depends on the electronic traps, especially the dangling bonds, in the interface between the QD core and its coating, which must be eliminated. This is usually done by a passivation layer of a wider optical band gap material [3]. The next important step is to functionalize the highly fluorescent QDs to specifically bind to recognize molecules such as proteins, peptides and nucleic acids, forming bioconjugates [4].

QDs show a number of advantages when compared to usual fluorescent dyes. Resch-Genger and colleagues indicated the high photostability as the most important advantage of QDs in comparison to organic dyes, showing almost no photobleaching [5]. This characteristic allows QDs’ fluorescence to be followed during biological processes, facilitating the 3D reconstructions and fluorescence quantification [6,7]. Other important optical advantage is the broad excitation spectrum, from UV to the optical band-gap. This means that one laser line can excite several QDs fluorescence bands, differently from usual fluorophores that require one specific excitation laser for each emission band. Moreover, QDs emission bands are brighter and narrower compared with conventional dyes [6,8,9], which allows the visualization of more specific biological structures.

**Figure 1.**
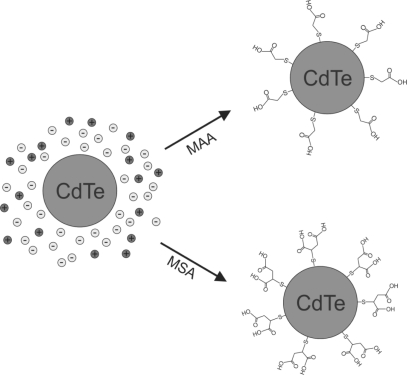
Method of CdTe passivation and functionalization with MAA [3], or MSA. (Adapted from Khatchadourian *et al*., 2007 [25]).

QDs can be capped with chemically inert materials such as silica to protect the cell and the QD, avoiding both cell cytotoxicity, and chemical and/or metabolic degradation of the QD labeling inside the cells [10]. Moreover, QDs passivation or functionalization with thiol molecules [11–13] can be used as a universal bioconjugation. For example, mercaptoacetic acid (MAA), and mercaptosuccinic acid (MSA), link the thiol (-SH) termination with the CdTe QD, forming a small passivation layer of CdS around the nanoparticle. On the other hand, MAA also acts as a functionalization layer due to the affinity of OH radical with terminal amino groups in proteins [3,14] (see Figure 1).

Previous studies demonstrated that QDs are more photostable than FITC (fluorescein isothiocyanate) for *Cryptosporidium parvum* [15]. The FITC and other conventional fluorochromes have been extensive used in pathogenic protozoa including *Trypanosoma cruzi* for different purposes [16–18]. Among the applications, it was employed for infection determination or for localization of specific antigen on the parasite using antibody conjugated fluorescent markers. Antibody conjugated QDs are also used in the same context [19–21]. Tokumasu *et al*. used QDs to evidence the interaction of erythrocytes infected with *Plasmodium falciparum* and the innate defense of the vertebrate host [8]. Subsequently, investigations about the parasite-vector interaction was performed by our group in *T. cruzi* epimastigotes and *Rhodnius prolixus* model [22], using CdTe QDs synthesized in our laboratory, as previously published [23,24]. Our choice of thiol capped QDs was based on the simple functionalization procedure described above, and also, based on low costs, synthesis, simplicity and flexibility. For instance, the MAA or MSA thiol group can be replaced during the production of QDs to another molecule that best fits the application, a desirable characteristic for specific biolabeling.

Table 1 shows some studies of QDs marked microorganisms or viruses, including QDs uptake and its toxicity. Some studies have evidenced the low QD’s cytotoxicity was observed in different cellular models [10,26,27]. These studies are fundamental for the validation of the biological and medical applications [28].

## 2. QDs Uptake by the Cells

Endocytosis is a crucial mechanism that eukaryotic cells use to internalize extracellular material, required for developmental processes in metazoans and unicellular organisms [29]. The uptake of nutrients by the cells can be classified by the particle size as: (a) phagocytosis—where large size particles are engulfed; (b) macropinocytosis (>1 μm); (c) clathrin-mediated endocytosis (120 nm); (d) caveolin-mediated endocytosis (60 nm); (e) caveolin and clathrin independent endocytosis (90 nm) [30,31]. It has been shown that the internalization of nanoparticles is size dependent and mediated by a receptor, with an optimal radius of approximately 25 nm [32,33]. However, one must take into account the differences between labeling of live or “fixed” cells. The use of chemical components that stabilize proteins and lipids led to the formation of pores that will facilitate the QDs entry [34].

Previous reports showed the internalization of QDs conjugated to human transferrin in live HeLa cells after 18 h incubation. The co-localization of QDs with specific membrane protein reinforces the participation of the endocytosis in the QDs entry into the cell [10], leading to an intracellular accumulation in the endocytic vesicles [35].

Moreover, *Dictyostelium discoideum* and HeLa cells incubated with QDs at low temperature (4 °C) were not stained, strongly suggesting the endocytic pathway as a mechanism of QDs uptake [10]. However, QDs internalization also depends on many factors such as the size of the nanoparticle and the functionalization layer [36]. The nanoparticles capping with PEG (polyethylene glycol) prevents the process of QDs aggregation in the cytosol and their cytotoxicity [36–38]. Anas and co-workers showed that peptides QDs conjugated are internalized by clathrin-mediated endocytosis in human A431 epidermal cells and mouse 3T3 fibroblasts cell lines [35]. The uptake and intracellular transport of QDs bioconjugated by live HeLa cells have been investigated systematically by confocal microscopy [39]. It was shown that QDs were predominantly internalized by clathrin-mediated endocytosis, or macropinocytosis, in a few cases. These authors also observed QDs vesicles being actively transported along microtubules towards the perinuclear region and into lysosomes, in contrast to other nanoparticles that suffered exocytosis or have been found in the cell periphery [39].

## 3. QDs Internalization in Microorganisms

### 3.1. Bacteria

QDs internalization by prokaryotes, so far, has not been well-understood. In principle, an endocytosis internalization process should be excluded because this is an eukaryote specific process, that has never been reported for prokaryotes, although there is a 2010 report that observed endocytosis in a specific bacterium [40]. Some authors proposed three possible mechanisms through which nanocrystals could pass through prokaryotic cell wall: (a) nonspecific diffusion; (b) nonspecific membrane damage; and (c) specific uptake. Nonspecific diffusion is unlikely because “*the mean estimate of the effective hole radius in walls from E. coli is 2.06 nm*” (2.12 nm for *B. subtilis*) [41], is smaller than a typical 3 to 4 nm radius QD [42]. Entry of other types of nanocrystals into bacteria via nonspecific membrane damage has been established. Previous studies points to the size of nanoparticles relating to its redox potential upon light exposure and electron transfer between QD and the surrounding water-based medium. It would lead to free radicals release, which causes transient membrane damage, allowing the particle entry. Specific transport is another possibility, because the pore sizes are larger than those for nonspecific diffusion. The largest pores known are associated to bacteria exocytosis and its openings reach up to 6 nm [43].

### 3.2. Fungi

In yeast, the best characterized pathway for endocytosis depends on the clathrin coated vesicle [44]. The hallmark events of endocytic pathways in these organisms include plasma membrane invagination followed by the pinching off the invaginated endocytic vesicle. Several adaptor proteins are involved in the endocytosis and exocytosis and were important for growth, differentiation and/or virulence [45]. In *Saccharomyces cerevisiae*, Pan1 is an endocytic protein that regulates membrane trafficking, actin cytoskeleton and signaling. The molecular mechanism of endocytic vesicle formation has been extensively examined in this model [46]. Shaw and colleagues [47] demonstrated that the heavy and light chains of the coat protein clathrin, and its adaptor protein Ede1, are the first proteins recruited to the endocytic site in yeast [48]. In *Cryptococcus neoformans*, a multi-modular endocytic protein, Cin1, was recently found to have pleiotropic functions in morphogenesis, endocytosis, exocytosis and virulence [48]. Although QD labeling was employed to monitor molecules uptake, such as glucose, in *S. cerevisiae*, since 2006, the intracellular trafficking of these nanoparticles [13–49] was not elucidated.

### 3.3. Virus

Viruses with diameters between 20 to 300 nm are not much bigger than 4 to 10 nm QDs. Therefore, internalization of QDs inside the virus is not possible. However QDs can bind specifically to virus’ proteins and be used to observe the internalization of the whole virus-QD complex inside the cells. The crucial steps in establishing a successful virus infection are critical factors such as attachment and internalization into a host cell as well as the determination of the tropism species. During the entry, viruses use various cellular structures to optimize the delivery of its genome to the nucleus promoting an efficient viral replication. The understanding of the viral transport requires the elucidation of molecular interactions between the virus and target cells, but unfortunately these mechanisms are still poorly understood [50]. Several endocytic pathways are exploited by viruses, including clathrin- and caveolin-dependent or independent endocytosis [51]. However, only few studies have investigated the mechanism of internalization of QDs labeled viruses. Joo and co-workers monitored the intracellular dynamics of Adeno-Associated Virus serotype 2 (AAV2), a small nonenveloped virus, that belongs to the family of parvoviruses, labeled with QD (QDs 525 or 705 ∼10 nm diameter, commercially available at Invitrogen) [50]. AAV2 was internalized mainly through a clatrin-dependent pathway and trafficked through endosomes and the cytoskeleton network. It was also observed that QD-labeling procedure did not affect the AAV2 infection. Recently, Liu and colleagues demonstrated that the Hematopoietic Necrosis Virus (IHNV), an important fish pathogen that infects salmonids, is a valuable model system for exploring the host entry mechanisms of virus [52]. A long-term tracking of IHNV entry was evaluated by QDs labelling and showed that IHNV is internalized through clathrin-coated pits [52].

### 3.4. Protozoa

**Figure 2.**
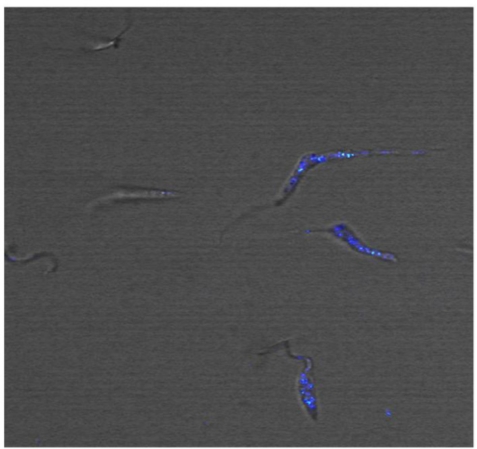
Live parasites imaging using QDs bioconjugates with lectin (*Sambacus Agglutinin Nigra*—SNA) (After Feder *et al*. 2009 [22]).

In trypanosomatids that are important pathogens transmitted by insect vectors, the endocytosis process is well investigated. There are three model organisms that have been most extensively studied: *Trypanosoma brucei*, the causative agent of African sleeping sickness, *Trypanosoma cruzi*, responsible for Chagas’ disease, and parasites of genus *Leishmania sp.*, which cause the different clinical forms of leishmaniasis [53–55]. Exclusively in *T. cruzi* epimastigotes, the flagellar pocket and cytostome are the surface domains involved in acquisition of nutrients. The labeling of live promastigotes of *Leishmania amazonensis* using CdS/Cd (OH)_2_ nanoparticles functionalized with polyphosphate anions and/or glutaraldehyde molecules was reported in 2006 [49]. Although the authors could distinguish organelles and structures by a dual fluorescence emission, the labeling process is not yet fully understood. Later, Chaves and co-workers described the application of CdS QDs in epimastigotes [27]. Transmission electron and fluorescence microscopy analysis pointed to the labeling with QDs in the cytostome, as well as the reservosomes, organelles that storage molecules and nutrients in the parasite. Recently, our group showed vesicles in parasites labeled with QDs bioconjugated with lectin-SNA (*Sambucus nigra agglutinin*) that have specificity to α-NeuNAc-Gal, α-NeuNAc-GalNAc, and, to a lesser extent, α-NeuNAc-Gal residues, suggesting that these QDs were internalized by endocytosis (see Figure 2) [22]. A blockage of the endocytosis by incubation with QDs at 4 °C was also observed even after extended periods (>2 h). Despite the fact that some studies have shown the entry of QDs in epimastigotes, the elucidation of the internalization route of QDs remains obscure in *T. cruzi*, and our group is performing further investigations to clarify this process.

## 4. QDs’ Cytotoxicity

QD toxicity has been extensively studied in various prokaryote and eukaryote models. Besides the advantages of using nanomaterials such as QDs in the biomedical area, many studies have been conducted to evaluate the possible toxicity of these nanoparticles [56–58]. The cytotoxicity of QDs can be linked to the photochemical process resulting from its irradiation under aerobic conditions *in vivo* [59,60]. The occurrence of photo oxidation of CdTe in *Euglena gracilis* (EG 277) and human embryonic kidney (HEK 293) cells have been observed [61]. The photobleaching for the cellular QDs is dependent both on the irradiation power density and the QD local concentration. The higher irradiation power density, oxygen abundance and lower QD concentration will result in a higher photobleaching rate [60–62].

This process seems to involve a transfer of an electron to an excited QD O_2_ molecule that produces a superoxide anion, which may lead to rust and corrosion of the surface of the nanoparticle [63]. Excited QDs can also transfer energy to neighboring molecules by a process called Energy Transfer leading to formation of reactive singlet oxygen species [56–64]. Concomitantly, in the case of QDs made with cadmium, cytotoxicity is a consequence of the release of free cadmium ions (Cd^2+^) that are highly toxic [57]. Cadmium (Cd), selenium (Se) and tellurium (Te) (CdSe and CdTe) are toxic elements for humans, causing toxicity in the kidneys, lungs, nervous system, in addition to DNA damage [65,66]. Any interaction of nanoparticle with some exogenous factor that leads to the segregation of these atoms can trigger the release of Cd and Te or Se in the cells. A mechanism that reduces the QDs toxicity is the synthesis of a passive layer, since the nanoparticle is protected from interaction with the cellular environment [36]. It has been shown in the literature that cells containing QDs can maintain their biological functions active, remaining viable for several generations [67]. Jaiswall and colleagues observed that the cells remained in good condition, continuing the process of QDs endocytosis at least until 20 days after incubation with the nanoparticles [10].

One thing important to mention about the toxicity of QDs is that it is difficult to generalize results in various models. Several types of QDs capped were tested in different ways in distinct cell types. The chemical composition and passivation of the nanoparticle is directly related to toxicity in cells [68]. According to Pelley and colleagues, each result is useful in guiding to a new experimental protocol for the toxicity tests. Few studies specifically focused on experiments in cell toxicity of QDs, in relation to dose, duration, frequency of exposure and mechanisms of action [58]. Hoshino and co-workers observed that the toxicity of QDs appears to be dose dependent [36]. Previously, it was shown that 10 μg/mL CdTe QDs capped MPA and cysteamine was cytotoxic in PC12 cells (cell line derived from a pheochromocytoma of the rat adrenal medulla) [69], but this same work indicated a toxicity of non-capped CdTe at a concentration of 1 μg/mL [69]. CdTe QDs cytotoxicity have been studied in mammals and microorganisms such as bacteria, protozoa, fungi and viruses [10,13,50,59,70].

## 5. QDs Toxicity for Bacteria

Wang and co-workers evaluated the toxicity of a series of QDs compositions, namely CdSe, CdTe and ZnS-AgInS_2_ (ZAIS), against luminous bacteria (*Photobacterium phosphoreum*) as a microbial sensing element [71]. They show that different coatings, such as MAA, dihydrolipoic acid (DHLA), among others, induced changes that were responsible for cytotoxicity. Previous studies have attributed bacterial toxicity mechanisms to the photogeneration and ROS formation. The phototoxicity generated by sunlight and high intensity lamps cause the direct release of metal ions (e.g., Cd^2+^) [59,72]. The susceptibility of Gram-positive and Gram-negative strains (*Pseudomonas aeruginosa* (American Time Culture Collection 10145U), *Escherichia coli* (ATCC25922), *Staphylococcus aureus* (ATCC29213) and *Bacillus subtilis* (ATCC9372) to QDs is very debatable [73,74]. It has been suggested that the differences in the sensitivity between the strains can be attributed for the sporulation process as well as other characteristics of cell wall, that may interferes with QDs binding, leading to ROS generation or the direct oxidation of cell lipids and proteins [75]. Other possibility is that the interaction of metal or nanoparticles with components of electron transport chain may cause toxicity by the inhibition of the respiration [76], but the resistance of Gram-positive and Gram-negative to the heavy metal is very different. The heavy metal damage was observed in *Bacillus subtilis* incubated with CdSe, however these microorganisms have an extrusion mechanism that enable Gram-positive bacteria to pump out Cadmium ions [77]. Although secondary effects could be detected, such as the membrane depolarization, the most important antibacterial effect of QDs depends on hydroxyl and superoxide radicals produced by the photo-generation of reactive oxygen species [78].

## 6. QDs Toxicity for Fungi and Virus

Only few studies were performed about the toxicity of QDs in fungi and virus. QDs presented low cytotoxicity for living yeast cells in comparison to other fluorescent markers [13,49]. For viruses, no investigations about the QDs toxicity were developed yet.

## 7. QDs Toxicity for Protozoa

Our group investigated the mechanisms of toxicity of CdTe QDs in *Trypanosoma cruzi*, the protozoan that causes Chagas’ disease. We found that nanoparticles at concentrations below 20 μM did not affect the integrity, motility and cell division of parasites [70]. Cell cycle assays demonstrated a dose-dependent increase in DNA fragmentation induced by the incubation with 2–200 μM CdTe QDs, also reduction in the percentage of DNA duplicated parasites.

**Figure 3.**
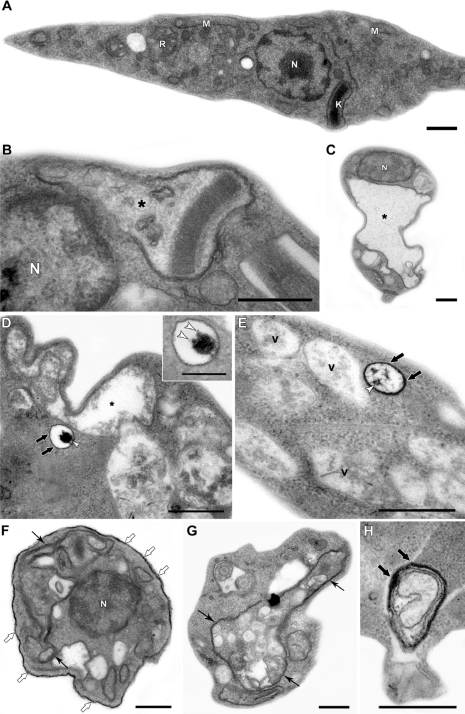
Ultrastructural effects of CdTe QDs on *T. cruzi* epimastigotes. (**A**) Untreated epimastigote showing normal morphological characteristics; (**B**–**H**) Parasites incubated with 200 μM QDs presented mitochondrial swelling (black asterisks) and the cytosolic vesicles containing QDs (thick black arrows). Aggregates of QDs inside the structure were observed (white arrowheads). The incubation of QDs also induced intense vacuolization in the cytosol (V) and the appearance of endoplasmic reticulum profiles (black arrows) surrounding organelles and other cytosolic degraded structures. QDs were also detected on the epimastigote surface (thick white arrows). Nucleus (N), mitochondrion (M), kinetoplast (K) and reservosome (R). Bars: A–H: 0.5 μm; inset in D: 0.2 μm.

Figure 3, the ultrastructural analysis of epimastigotes incubated with 200 μM QDs, shows: (a) intense vacuolization in the cytosol; (b) mitochondrial swelling; (c) the appearance of endoplasmic reticulum profiles surrounding organelles and other cytosolic degraded structures. The presence of the nanoparticle could be observed inside the cytosolic vesicles and also on epimastigote surface. Stahl and co-workers (2011) also observed that parasites incubated with 2 μM CdTe QDs presented the integrity of cellular organelles such as mitochondria, nucleus, Golgi apparatus and plasma membrane similar to the untreated groups [70]. Such concentration was sufficient to label the epimastigotes and was not toxic, at least up to 72 h, reinforcing the possibility of several applications of QDs in these parasites. We also described several morphological alterations, in a previous study, especially indicative of autophagic process. This raised the possibility that high concentrations of QDs on the parasite surface could lead to autophagy by an unknown pathway, a question that has not been answered yet. The observation of QDs in membrane vesicles and extensive changes in the plasma membrane near the flagellar pocket region suggests a membrane shedding, without any disorganization in the subpellicular microtubules cage [70]. Some membrane blebbing induced by QDs was also described in bacteria [59].

## 8. Conclusions

The toxicity of QDs is associated with their physicochemical properties. Due to the large diversity of nanoparticles (CdTe, CdSe, CdS, CdS/Zn, PbSe, PbTe and others) and different capping techniques (MAA, zinc sulfide, MAS) employed, it is not possible to elucidate all the toxicity mechanisms. The concentrations used in each model vary greatly. Several attempts have been made to reduce particle size by (a) selecting the capping of the nanoparticles; (b) using minimal doses; and (c) modulating nanoparticle size [11,68,79,80]. All these factors are important for cell toxicity response and consequently to the use of QDs as fluorescent marker. As described in the literature, the major QDs toxicity is relate to induction of reactive oxygen species (ROS) formation or the direct release of metal ions (e.g., Cd^2+^). In most cells, these reactions cause cellular changes culminating in DNA damage. However, some biological questions remain open and need to be answered in order to optimize the use of QDs in the target cell.

## Figures and Tables

**Table 1.** QDs on different microorganisms (ND—Not Determined).

**QDs**	**Capping layer**	**microorganisms**	**concentration**	**localization**	**Toxicity**	**Refs**
CdTe-CdS (600 nm)	Mercaptoacetic acid (MAA)	Living yeast cells (HEBRON)	Glucose/CdTe–CdS (1:5)	Cytoplasm	Low cytotoxicity	[13]
CdZnS/CdSe (manufatured)	Mercaptoacetic acid (MAA); 1-ethyl-3-(3-dimethylaminopropyl) carbodiimide hydrochloride (EDC)	Gram positive and negative bacteria (*P. aeruginosa* (ATCC 10145U), *E. coli* (ATCC25922), *S. aureus* (ATCC29213) and *B. subtilis* (ATCC9372)	0.05–0.2 μM	ND	Metabolic damage	[43]
QDs 525 nm/QDs 705 nm (Invitrogen)	1-ethyl-3-(3 dimethylaminopropyl) carbodiimide hydrochloride (EDC)	Adeno-associated virus (AAV 2)	30 pmol of carboxyl QD conjugated	Endosomes	No toxicity	[50]
Cd Te (manufatured)	3-mercaptopropionic acid (MPA)	Bacterium (*E. coli*, *B. subtilis, P. aeruginosa* and *S. aureus*)	0.25 for 0.3 μm CdTe	Intracellular compartments	Membrane blebbing, emptiness cytoplasm and reactive oxygen species (ROS) formation	[59]
CdTe (560 nm)	Mercaptoacetic acid (MAA)	*Trypanosoam cruzi*	0.2–200 μM	Cytosolic vesicles	DNA fragmentation; membrane blebbing; mitochondrial swelling and cytosolic degraded structures.	[70]
CdTe/CdSe (manufatured)	Mercaptoacetic acid (MAA)	Luminous bacteria (*Photobacterium phosphoreum*)	0.1 g/L	Bacterial surface	Changes of surface and bacteria’s compounds	[71]
CdTe Qds (manufatured)	ND	Gram positive and negative bacteria (*E. coli* and *B. subtilis*)	1.2 nM	ND	Reactive oxygen species (ROS) formation	[78]
QDs CdSe 525/655 nm (streptoavidin conjugated)	ND	Human immunodeficiency virus (HIV)	20 nM	Virus surface	ND	[81]
QDs 525/655 nm (streptoavidin-conjugated invitrogen)	ND	Hepatitis A virus (HAV)	10–50 nM	Mammalian enteric cells surface	ND	[82]
Cd Te (Aldrich)	TGA capped (tripepthide glutathione); CSH capped	Bacterial strains *Cupriavidus metallidurans* CH34; *C. metallidurans* AE104; *E. coli* MG1655; *Shewanella oneidensis* MR-A; *B. subtilis* LMG 7135T	1 μM	ND	Changes of bacteria’s morphology	[83]
CdS/Zn Se 655/585 nm (invitrogen)	ND	*Bacillus anthracis* and *Yersinia pestis*	4 μM	ND	ND	[84]
